# One or two ligatures inducing periodontitis are sufficient to cause fatty liver

**DOI:** 10.4317/medoral.22204

**Published:** 2018-04-24

**Authors:** Larissa-dos Santos Pessoa, Felipe-Rodolfo Pereira-da Silva, Even-Herlany-Pereira Alves, Luiz-Felipe-de Carvalho França, David di Lenardo, Joaquina-dos Santos Carvalho, Victor-Brito-Dantas Martins, Francisca-Beatriz-de Melo Sousa, Karina-Oliveira Drumond, Jand-Venes-Rolim Medeiros, Jefferson-Soares de Oliveira, Daniel-Fernando-Pereira Vasconcelos

**Affiliations:** 1 Laboratory of Histological Analysis and Prepare (LAPHIS), Federal University of Piaui, Parnaiba, PI, Brazil; 2 Laboratory of Experimental Physiopharmacology (LAFFEX), Federal University of Piaui, Parnaiba, PI, Brazil; 3 Laboratory of Biology and Biochemistry Plants (BIOqPLANT), Federal University of Piaui, Parnaiba, PI, Brazil

## Abstract

**Background:**

Periodontitis is a chronic disease that due to an intense inflammatory response triggers systemic changes such as hepatic alterations. This study aimed to compare hepatic damage in rats that received experimental periodontitis at one or two periodontal sites with ligatures.

**Material and Methods:**

Eighteen rats were separated into three groups: control, without ligature; periodontitis 1, with one ligature; and periodontitis 2, with two ligatures. The following parameters were assessed: gingival bleeding index, probing pocket depth, tooth mobility, alveolar bone loss, malondialdehyde (MDA) and myeloperoxidase (MPO) activity in periodontal tissue; histopathological evaluation of hepatic tissue (steatosis score); glutathione levels (GSH), MDA, MPO, cholesterol and triglycerides in the liver; and serum levels of alanine aminotransferase (ALT) and aspartate aminotransferase (AST).

**Results:**

Periodontal evaluation data showed that the periodontitis model worked well. The groups with periodontitis did not differ significantly in relation to MPO activity and MDA levels in the gingival samples, but they were significantly different when compared with the control group. Steatosis was observed in the histological analysis of the groups with periodontitis, but between the periodontitis groups, two ligatures did not cause increase in steatosis score. The levels of GSH, MDA, total cholesterol and triglycerides in the hepatic tissue were not altered between groups with periodontitis, but they showed significant differences in comparison with the control group. The activity of MPO in hepatic tissue and serum levels of AST and ALT did not present significant difference among the three groups.

**Conclusions:**

In conclusion, our results demonstrated that one or two ligatures inducing periodontitis were both sufficient to cause fatty liver. Steatosis caused by two ligatures did not present larger extension and severity than steatosis caused by one ligature.

** Key words:**Antioxidant, anti-inflammatory agents, experimental design, periodontal disease, periodontal medicine.

## Introduction

Periodontitis is a chronic inflammatory disease (1) caused by the presence of a bacterial biofilm composed of several species, such as *Porphyromonas gingivalis, Treponema denticola, Prevotella intermedia,* and *Fusobacterium nucleatum* (2) that affect periodontal tissues, besides the response of the host against these microorganisms (1). It can trigger systemic alterations such as cardiovascular diseases (3-4) impaired renal function (5-6), structural changes and mainly hepatic damages (7).

Although several studies have been published associating periodontitis with changes in the liver (8), the mechanism by which this process occurs is not yet well understood (8-9). Some studies have demonstrated high levels of inflammatory markers in the blood in patients with periodontal disease (10). This event results from combination of the host response with the proliferation of the pathogens and their products (lipopolysaccharides - LPS - and proteases) (11), which triggers intense ulceration of the gingival region (8), allowing the dissemination of microorganisms in the bloodstream.

Epidemiological studies have demonstrated that patients with periodontitis present a serum increase of aspartate aminotransferase (AST) and alanine aminotransferase (ALT), which are important markers of liver injury. Alterations of these markers have also been observed in individuals with steatosis (8-12).

Non-alcoholic steatosis (NAS) is the most common form of chronic liver disease and is characterized by accumulation of fat in hepatocytes (13). Steatosis can be considered a multisystem disease associated with inflammation, overproduction of reactive oxygen species (ROS) produced by lipid peroxidation such as malondialdehyde (MDA), and reduction of antioxidant molecules such as glutathione (GSH). Levels of these molecules are normally measured in altered liver tissues for evaluation of oxidative stress (14). The inflammation and oxidative stress affect adipose tissue and can result in insulin resistance showing an association of periodontitis with metabolic syndrome (3).

The rat model has been the most used to induce periodontitis. Studies have shown systemic damage associated with induced periodontitis in rats (6-14), especially hepatic lesions. We hypothesized that the increase in the number of periodontitis induction sites can aggravate hepatic damage. In the literature there are no studies showing this association. Thus, this study aimed to assess whether liver damage is associated with the number of sites injured by ligatures in an experimental periodontitis model.

## Material and Methods

- Animals

In this study 18 female Wistar rats (Rattus norvegicus) weighing 188.6 g were used. They were kept at a temperature of 23 ± 2 °C, with a light/dark cycle of 12 hours, and free access to water and food. The animals were later euthanized according to the recommendations of the Guide for Care and Use of Laboratory Animals (National Institute of Health, Bethesda, MD, USA) and the guidelines of the Institutional Animal Ethics Committee (protocol number 385/17).

- Experimental design

Rats were randomly divided into three groups of six: control (no ligature, both right and left first molars were evaluated and the mean value was used), periodontitis 1 (right first lower molar with one ligature, for 20 days, where only the ligated tooth was evaluated), and periodontitis 2 (both lower molars with ligatures, for 20 days, were evaluated and the mean value was used in the statistics for the periodontal parameters). The periodontitis was induced under general intramuscular anesthesia by injection of a solution of 35 mg/kg of ketamine (Francotar-Virbac, Roseira, SP, Brazil) and 15 mg/kg of xylazine (Rompum-Bayer, São Paulo, SP, Brazil). A nylon ligature (3-0, Shalon, Goiania, GO, Brazil) was placed around the cervical region of the right lower first molar in the group with one ligature (11) and in the group with two ligatures, these were placed around both lower first molars. Blood samples for biochemical tests were collected from all 18 rats, from the retro-orbital plexus. Then the rats were sacrificed and their body and liver weights were measured.

- Gingival bleeding index (GBI)

First molars were probed in the region of the periodontal pocket or gingival sulcus for ten seconds using a probe with a radius of 0.2 mm, and the classification of the GBI as described by Liu *et al.* (15) was performed according to scores from 0 to 5: score 0: gingival margin and gingival papilla (GMP) are healthy; score 1: GMP mildly inflamed no bleeding; score 2: GMP mildly inflamed; changes in color, absence of edema, punctate hemorrhage; score 3: GMP moderately inflamed; changes in color, mild edema, and bleeding in the gingival crevice; score 4: GMP severely inflamed; changes in color, severe edema, with blood flowing out of the gingival crevice; score 5: GMP severely inflamed; changes in color, severe edema, ulceration, and spontaneous bleeding, and blood, with blood observed flowing out of the gingival crevice.

- Probing pocket depth (PPD)

It was used a periodontal probe (0.2 mm radius) on first lower molars. Three points were measured: mesio-buccal, disto-buccal, and mid-buccal, and the PPD mean values were obtained (15).

- Tooth mobility 

The evaluation of the tooth mobility was performed by scoring as described by Xu *et al.* (16). The mobility of the first right and left first molars was classified as follows: 0 = physiological mobility, 1 = slight mobility (vestibular-palatal), 2 = moderate mobility (buccal and mesial-distal) and 3 = severe mobility (tooth moves in and out of the socket).

- Measurement of alveolar bone loss (ABL)

**Figure 1:**
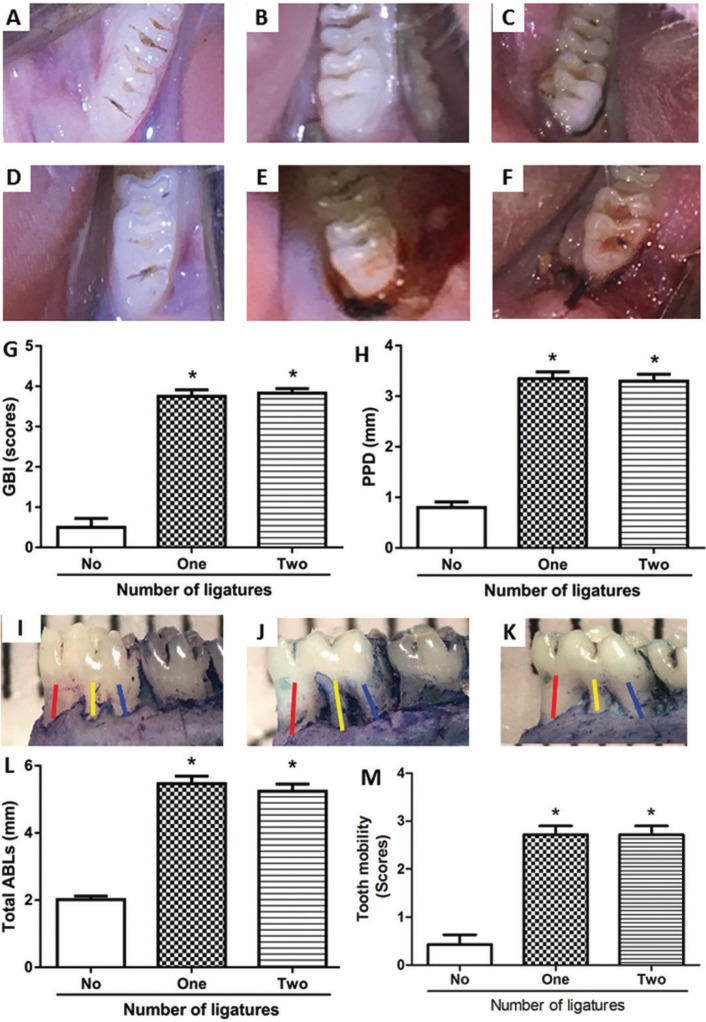
A) and D) depict the clinical analysis of the control group. B) and E) clinical analysis of the periodontitis group with one ligature. B) showing the left side without ligature and without changes. E) right side showing intense inflammation, with color change, edema and bleeding after probing. C) and F) clinical analysis of the periodontitis group with two ligatures. C) showing the left side with inflammation, edema, color change, without bleeding. F) right side showing intense inflammation, edema and bleeding after a slight probing. G) GBI, showing high values for groups with periodontitis when compared to the control group. The comparison between groups with ligature did not present significant changes. H) PPD, showing increase for groups with periodontitis in relation to the control group. There were no significant changes between groups with ligature. I), J) and K) depict the clinical difference of the alveolar bone. I) in control, without change. J) and K) groups with one and two ligatures presenting alveolar bone loss (Red, yellow and blue lines indicate ABL-1, ABL-2 and ABL-3, respectively). L) ABL, demonstrating the significant difference of the groups with periodontitis in comparison with the control. M) tooth mobility, showing a significant difference of the groups with ligature in relation to the control. * *p*<0.05 indicates periodontitis groups versus control group.

The ABL was measured to demarcate the cement-enamel junction (JEC) of the first lower molars. After dissection of the soft tissue, the mandibles were impregnated with aqueous solution of 1% methylene blue. The image of the alveolar bone height for each hemimandibula was captured using a stereomicroscope at magnification of 30x. For evaluation of the alveolar bone, three points of the lingual region were measured. The measurements were made along the root axis, as follows: a) ABL-1, the distance was ascertained by measuring the height of the cement-enamel junction to the alveolar crest, at the mesial root of the first lower molars; B) ABL-2, the distance was obtained by measuring the height of the cementum-enamel junction to the alveolar crest, at the intermediate root of the first lower molars; C) ABL-3, the distance was determined by measuring the height of the cementum-enamel junction to the alveolar crest, at the distal root of the first lower molars (Red, yellow and blue lines in the figure 1 indicate ABL-1, ABL-2 and ABL-3, respectively). After analyzing the three jaw points, the following formula was used: ABL = ABL-1/3 + ABL-2/3 + ABL-3/3. Images were evaluated by image an analysis system (ImageJ v.1.48 Media Cybernetics, ImageJ v.1.48 Software).

- Concentration of malondialdehyde (MDA) in the liver and gingival tissue

The levels of MDA in liver samples were determined according to the method described by Mihara and Uchiyama (17), which is often used to estimate lipid peroxidation. This method is based on the extraction of this compound using an organic solvent (n-butanol), followed by measurement of the MDA concentration.

- Myeloperoxidase (MPO) activity in the liver and gingival tissue

Analysis of MPO activity was performed to determine the infiltration of neutrophils in the gingival tissue around the both first molars, as described by Chaves *et al.* (18). For this purpose, 45 mg of gingiva was homogenized at 55 mg/mL in potassium buffer containing 0.5% hexadecyltrimethylammonium bromide (HTAB). The MPO activity was assessed by the difference in absorbance at 450 nm with the use of o-dianisidinedihydrochloride and 1% hydrogen peroxide.

- Levels of hepatic glutathione (GSH)

The level of GSH in liver samples was determined as described by Sedlak and Lindsay (19) and was determined by observing the reaction of the sulfhydryl group with 5,5 ‘-dithiobis (2-nitrobenzoic acid) (DTNB-Ellman’s reagent) with the free thiol, which results in a mixture of disulphide plus 2-nitro-5-thiobenzoic acid. The resulting product was measured by spectrophotometry.

- Histopathological assessment of liver tissue 

Samples were stored in 10% buffered formaldehyde. The histological processing of the samples followed the following steps: dehydration, immersion in increasing solutions of ethyl alcohol; diaphanization, xylol solution; inclusion, paraffin impregnation and cutting of sections with 6 μm thickness. The sections were stained with hematoxylin and eosin and toluidine blue for assessment of mast cell density; and periodic acid-Schiff (PAS) for glycogen evaluation (original magnification 150x). The PAS scoring was: 0: physiological glycogen accumulation; 1: light glycogen accumulation; 2: moderate glycogen accumulation; 3: severe glycogen accumulation. The slides were evaluated with light microscope (NOVA, Piracicaba City, SP State, Brazil). For histological analysis of the liver, images from each field of the histological slides were analyzed, as described by Turlin *et al.* (20) to determine the following parameters: a) steatosis, b) inflammation, c) necrosis, and d) mast cell density. The steatosis was evaluated semi-quantitatively by means of the analysis of the percentage of the cells with steatosis, following a scale of 5 degrees: 0, absent or present in <5% of hepatocytes; positive, ≥ 5% and <25%; grade 2, ≥ 25% and <50%; grade 3, ≥ 50% and <75% and grade 4, ≥ 75%. The evaluation of inflammation and necrosis used the following classification: 0: none; 1: <2 foci/field; 2: 2-4 foci/field; 3:> 4 outbreaks/field.

For the evaluation of mast cell density, the total number of cells was counted as revealed by toluidine blue, after which the number of cells was divided by the area found, obtaining the mast cell density per 2,500µm2 (21).

- Total cholesterol and triglyceride concentrations in hepatic tissue

Liver samples for determination of cholesterol and triglycerides were prepared using a 2: 1 concentration of chloroform and methanol respectively, and the resulting compound was used for tissue dosing. Samples of 100 mg were weighed in 1 ml of reagent. Then the macerated samples were centrifuged at 5,000 rpm for 6 minutes. The amount of supernatant used followed the recommendations of the kit’s manufacturer (Labtest, Belo Horizonte, MG, Brazil). The supernatant was homogenized together with the kit reagents. Subsequently, the samples with the reagents were analyzed by spectrophotometry (22).

- Serum levels of aspartate aminotransferase (AST) and alanine aminotransferase (ALT) 

The levels of hepatic injury markers (AST and ALT) were measured by colorimetry using biochemical kits (Labtest, Belo Horizonte, MG, Brazil) following the manufacturer’s instructions, and by spectrophotometric analysis.

- Statistical analysis

The results were expressed as mean ± SEM and/or median ± quartile interval. The Shapiro-Wilk test was used to check for normal distribution of the data. The differences between the groups were ascertained by analysis of variance (ANOVA). The Student-Newman-Keuls test was applied to parametric data and the Kruskal-Wallis test to nonparametric data, followed by the Dunn test for multiple comparisons. Differences were considered significant when *p* <0.05.

## Results

- GBI and PPD

The gingival papillas in the groups with ligature were inflamed, with changes in color, edema and bleeding after light probing (Fig. 1E,F). Both groups with ligature (Fig. 1B,C,E,F) when compared to the control group (Fig. 1A,D) presented clinical differences regarding the gingival tissue. There were no significant differences between the periodontitis groups 1 and 2 in relation to the GBI, but there was a significant difference when the two groups were compared with the control group (*P*<0.05) (Fig. 1G). In the PPD analysis, the means found for each group were 0.8 ± 0.1 mm for the control group, 3.3 ± 0.1 mm for the periodontitis group with one ligature and 3.3 ± 0.1 mm for the periodontitis group with two ligatures. There were significant differences regarding PPD between the control group and the groups with ligature (*P*<0.05, Fig. 1H). However, there were no significant differences between periodontitis groups 1 and 2 (Fig. 1H).

- Measurement of alveolar bone loss

The alterations observed in the alveolar bone of the control, one-ligature and two-ligature groups are represented in Figures 1I, 1J and 1K, respectively. There were significant differences between the groups with ligature in relation to the control group (2.0 ± 0.1 mm, 5.4 ± 0.2 mm, 5.2 ± 0.2 mm for control, one-ligature and two-ligature, respectively). However, there were no significant alterations between periodontitis groups 1 and 2 (Fig. 1L).

- Tooth mobility

No significant changes were observed between the periodontitis groups 1 and 2, but the comparison of these groups with the control group showed significant diffe-rences (*P*<0.05) (Fig. 1M).

Activity of MPO and levels of MDA in gingival tissue

The two groups with induced periodontitis, when compared to the control group, presented significant differences in relation to MPO activity (3.4 ± 0.8 U/mg of tissue, 51.4 ± 7 U/mg of tissue, 56.1 ± 7 U/mg of tissue, control, one-ligature and two-ligature groups, respectively; *P*<0.05), but the periodontitis groups 1 and 2 did not differ significantly between each other. In the comparison between periodontitis groups 1 and 2, MDA levels did not show significant differences, but the two groups had an increase in lipid peroxidation when compared to the control group (control with 37.8 ± 6.0 nmol /g of tissue, one-ligature with 79.2 ± 4.3 nmol/g of tissue and two-ligature with 77.3 ± 2.8nmol/g of tissue).

- Body and liver weight of animals

Comparison of the three groups in relation to liver weight did not show significant differences (*P*> 0.05) ( Table 1).

**Table 1:**
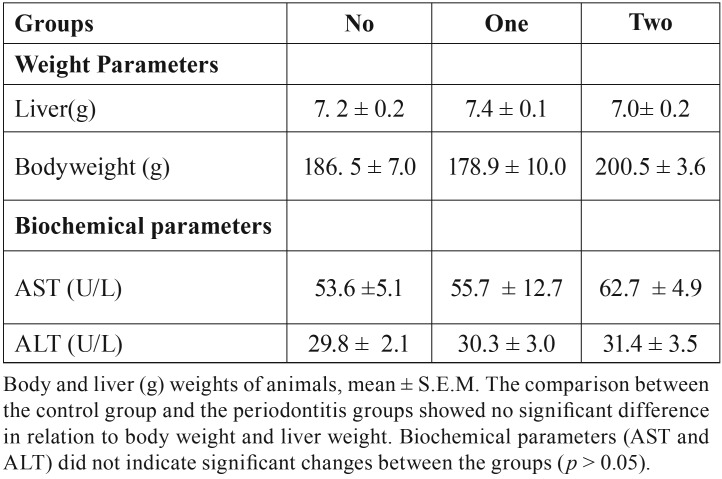
Body weight, liver and biochemical serum levels.

- Histopathological evaluation of the liver

**Figure 2:**
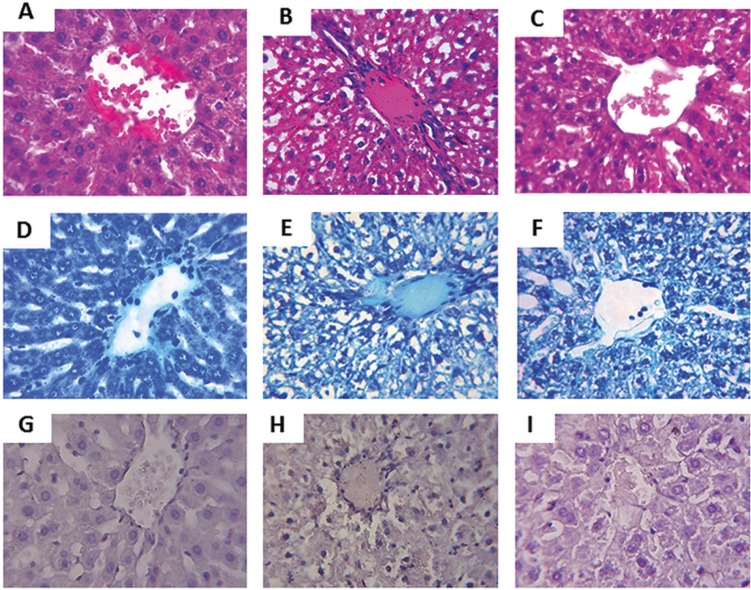
A) D) and G) depict the hepatic tissue of the control group without histological changes. Hepatocytes with normal conformation surround the central vein. B), E) and H) depict the hepatic tissue of the group with one ligature, showing hepatocytes with loss of conformation and steatosis. C), F) and I) depict the hepatic tissue of the periodontitis group with two ligatures. The hepatocytes exhibit altered cord conformation and presence of steatosis. (A, B and C) hematoxylin and eosin.(D, E and F) toluidine blue.(G, H and I) periodic acid-Schiff. Original magnification 150x.

Figures 2A, 2D and 2G demonstrate the hepatic tissue of the control group, in which the hepatocytes have normal conformation. The group with one ligature (Fig. 2B,E,H) and two ligatures (Fig. 2C,F,I) presented steatosis, with observation of some hepatocytes with steatosis of the microvesicular type and peripheral nuclei, besides a decrease of organization in the form of cords. Analysis of steatosis and PAS scores of the liver tissues of both groups with periodontitis showed significantly elevated steatosis index compared to the control group ( Table 2). However, periodontitis groups 1 and 2 did not present significant differences between them. The comparison of the three groups in relation to inflammation, necrosis score and density of mast cells did not show significant differences, as seen in the  Table 2.

**Table 2:**
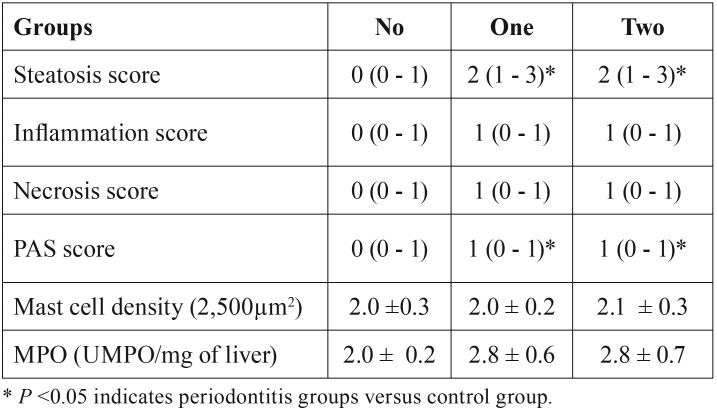
Hepatic parameters.

- MPO activity in hepatic tissue

MPO activity in the liver was analyzed to measure neutrophil infiltrate. No significant differences were observed between the groups in relation to MPO levels ( Table 2).

- Levels of GSH and MDA in liver

**Figure 3:**
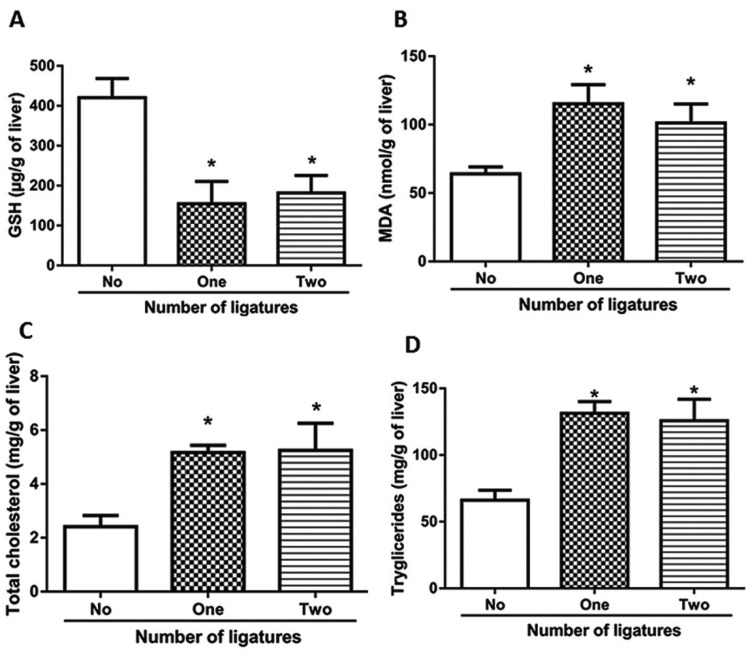
A) Depicts the levels of GSH in the liver. The control group presented a significantly higher concentration in relation to the groups with periodontitis. B) MDA concentrations in the hepatic tissue. The groups with periodontitis showed significant differences in relation to the control group. Cholesterol (C) and triglyceride levels (D) were higher in the periodontitis groups compared to the control group, but the comparison between them did not show significant changes.* *P*<0.05 indicates periodontitis groups versus control group.

The two groups that received induction of periodontitis demonstrated a significant decrease of GSH in the liver relative to the control group (420.7 ± 47.8 μg/g of tissue, 155.0 ± 55.7 μg/g of tissue, 181, 8 ± 43.7 μg/g of tissue in the control, one-ligature and two-ligature groups, respectively). However, there were no significant differences between periodontitis groups 1 and 2 (Fig. 3A). The concentrations of MDA in the groups with ligatures were higher compared to the control group (64.0 ± 5.1 nmol/g of tissue, for the control group, 115.3 ± 14.0 nmol/g of tissue, for the one-ligature group and 101.3 ± 13.8 nmol/g of tissue for the two-ligature group), presenting significant differences. Periodontitis groups 1 and 2 when compared did not present significant differences (Fig. 3B).

- Levels of total cholesterol and triglycerides in hepatic tissue

The liver samples of the groups with periodontitis had higher concentrations of total cholesterol than the control group (2.4 ± 0.4 mg/g tissue for the control, 5.1 ± 0.2 mg/g of tissue for one ligature and 5.2 ± 1.0 mg/g of tissue for two ligatures, *P*<0.05) (Fig. 3C), showing significant differences between them. However, the comparison between periodontitis groups 1 and 2 did not present differences (*P*<0.05). Triglyceride levels in the liver samples from the groups with ligature also showed a significant increase over the control group (control: 66.1 ± 7.5 mg/g of tissue; one ligature: 131.5 ± 8.7 mg/g tissue; two ligatures: 125.8 ± 16.1 mg/g of tissue) (Fig. 3D).

- Serum concentrations of AST and ALT

The AST and ALT values in the comparison of the three groups did not present differences (*P*> 0.05) ( Table 1).

## Discussion

The association between periodontitis and liver changes has been reported in the literature (23-24).Periodontitis can be associated with the development of steatosis, since the bacteria themselves or their products and cytokines released during inflammation can reach the circulation, similar to transient bacteremia (25), and then can reach the liver, leading to lipid peroxidation and oxidative stress, which are important factors involved with the development of steatosis (7-15).

One study demonstrated the presence of steatosis in models of periodontitis induced in rats with only one ligature (14). Our study is the first to report evaluation of steatosis in an induced model of periodontitis with the insertion of ligatures in both lower first molars, showing that two ligatures also caused steatosis, although the extent and severity were similar (Fig. 2). The histopathological evaluation of both groups with periodontitis presented degenerate and disorganized hepatocytes.

The data for the determination of periodontitis GBI, PPD, tooth mobility and ABL showed significant diffe-rences between the groups with periodontitis compared with the control group. The results of these parameters indicate the efficacy of the periodontitis induction model used, according to Vasconcelos *et al.* (14). Periodontitis confirmed through the above parameters allowed uniformly reliable analysis.

Due to the intense inflammatory process present in ligature-induced periodontitis, there is an increase of MPO activity in the gingival tissue (6,13,26). MPO is an enzyme present in neutrophils, commonly used as an indirect marker of neutrophil infiltration in inflammatory processes (26). Our results showed that MPO activity levels were higher in the groups with periodontitis compared with the control group, in agreement with the literature (6,13,26).This exacerbated inflammatory response results in the overproduction of reactive oxygen species, triggering lipid peroxidation and producing MDA as the product. The increase of this marker in gingival tissue has previously been observed in studies of periodontitis (6,27,28). Our results also demonstrate an increase in MDA levels in the groups with periodontitis in relation to the control group.

The liver disease known as steatosis occurs due to accumulation of lipids in hepatocytes. The accumulation of fat in liver cells can lead to degeneration and alteration in the conformation of these cells (14-28). In our study, these changes were observed in the histopathological analysis of the groups with periodontitis when compared with the control group. Histological comparison between the periodontitis groups with one or two ligatures did not show increase in the steatosis and PAS score. Indications of inflammation, necrosis and changes in mast cell density were not significantly observed in all groups. As observed previously (14), these parameters also did not change between the animals of the periodontitis and the control group.

In our study, despite the identification of inflammation in the liver samples, MPO activity did not present significant alterations between the studied groups. The absence of differences between groups regarding inflammation may explain the data on MPO activity in the liver. On the other hand, MPO activity levels are higher in hepatic tissue samples of obese individuals (29).We believe that the steatosis caused by periodontitis in rats is self-limiting, not advancing to steatohepatitis.

Oxidative stress causes changes in MDA and GSH levels in hepatic tissue (14). The MDA values were higher in the liver samples of the groups with periodontitis in relation to the control group, but the analysis between the groups with one ligature and two ligatures did not present significant differences. The oxidative stress observed in periodontitis induces a decrease in hepatic GSH, increasing oxidative imbalance and causing liver damage, according to previous studies (14,28).Comparison between periodontitis groups 1 and 2 did not show significant differences, similar to the MDA data, demonstrating that the changes caused in the liver by periodontitis were not directly associated with the number of sites damaged by the ligatures.

The liver plays a central role in lipid homeostasis. With liver damage due to periodontitis, lipid metabolism changes, resulting in increased levels of total cholesterol and triglycerides (28). In our study, total cholesterol and triglyceride concentrations of the groups with periodontitis were higher than in the control group, but did not have significant differences when compared to each other. ALT and AST are markers of liver injury that aid in the diagnosis of steatosis (24). In patients with steatosis, elevated levels of these markers in the blood are observed. Some studies with rats have shown no significant differences in ALT and AST values between periodontitis and control groups (14-28), as we also found. As can be observed, the data are still contradictory, so it is necessary to carry out new studies to better clarify the relationship of these markers with steatosis caused by periodontitis.

In addition, we assume that in clinical dentistry, patients with periodontitis can already present hepatic changes similar to those demonstrated by us. However, experimental periodontitis differs from that in patients, so the association must be extrapolated with caution.

In conclusion, our results demonstrated that one or two ligatures inducing periodontitis were both sufficient to cause fatty liver. Steatosis caused by two ligatures did not present larger extension and severity than steatosis caused by one ligature.
